# NK Cells in the Lymph Nodes and Their Role in Anti-Tumour Immunity

**DOI:** 10.3390/biomedicines12081667

**Published:** 2024-07-25

**Authors:** Lara V. Graham, Salim I. Khakoo, Matthew D. Blunt

**Affiliations:** School of Clinical and Experimental Sciences, Faculty of Medicine, University of Southampton, Southampton SO16 6YD, UK

**Keywords:** natural killer (NK) cells, lymph nodes, cancer, immunotherapy

## Abstract

The lymph nodes are vital to enable adaptive immune responses to infection. Natural killer (NK) cells are cytotoxic lymphocytes that directly kill cancer cells and modulate the activation of other immune cells during anti-tumour immune response. NK cells in the lymph nodes are involved in the regulation of T-cell and B-cell populations and the clearance of viral infections. In solid tumours, lymph nodes are a frequent site of metastasis and immune cell priming, whilst in haematological malignancies, tumour cells can proliferate in the lymph nodes. Thus, lymph nodes are an important site in anti-tumour immunity and therapy resistance. It is therefore crucial to identify strategies to increase recruitment and overcome suppression of NK cells in the lymph node microenvironment to improve tumour clearance. In this review, we summarise the literature interrogating NK cell phenotype and function in the lymph nodes in the context of infection and cancer and evaluate both current and potential strategies to mobilise and activate NK cells within the lymph nodes of cancer patients.

## 1. Introduction

**Figure 1:**
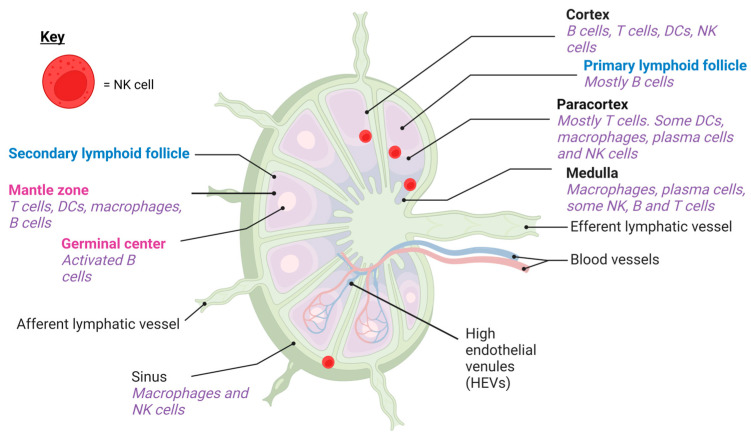
Distribution of NK cells in the lymph nodes. NK cells have been reported in the lymph node sinuses and paracortex of humans and the medulla, paracrotical and cortical areas of the lymph nodes in mice. Created with Biorender.com.

The lymph nodes are a critical part of the immune system which facilitate the interaction between innate and adaptive immune cells to elicit successful immune responses against pathogens [1]. In the resting lymph node of a healthy individual, the medulla, cortex and paracortex form discrete compartments [2], as well as sinuses through which lymph fluid flows as it enters and leaves the lymph node [3] (Figure 1). The medulla and paracortical areas contain T cells, B cells, natural killer (NK) cells, macrophages and plasma cells [2,4,5]. Dendritic cells (DCs), the main antigen-presenting cells in the lymph nodes, are present in the paracortex [6]. In the cortex, primary lymphoid follicles consisting of resting B cells and follicular DCs (fDCs) are observable, while T cells are generally found outside the follicles [2]. Lymph node stromal cells also have an important structural and immunoregulatory role [7]. For example, conduits formed by fibroblastic reticular cells lining the paracortical areas are important for distributing chemokines, cytokines and soluble antigens deep into the lymph node to facilitate immune responses [7]. Immune cells enter the lymph node through the afferent lymphatic vessels via the lymph fluid or from the peripheral blood through specialised blood vessels known as high endothelial venules (HEVs) that penetrate the lymph node structure [3]. B and T lymphocytes continuously circulate between the peripheral blood and lymphatic system until recognition of a foreign antigen occurs in the secondary lymphoid tissue, such as the lymph nodes, where they undergo rounds of selection, activation and proliferation to mount an antigen-specific immune response.

During infection, both naïve CD4+ and CD8+ T cells can be activated in the lymph nodes through interactions with antigen-presenting DCs. CD4+ T cells receive early activation signals from migratory antigen-presenting DCs in the lymph node, while CD8+ T cells receive support from lymph node-resident DCs, and this interaction is supported by activated CD4+ T cells [8]. After priming with antigen, activated CD4+ and CD8+ T cells differentiate into distinct subsets, which is controlled by specific transcription factors, cytokines, chemokines and interactions with B cells and DCs [9]. Activated CD4+ T cells can migrate to the B-cell primary follicles, and interactions with B cells and type 2 conventional DCs result in the upregulation of canonical T follicular helper (T_FH_)-associated genes [9].

The interaction between CD40L on T_FH_ cells and the CD40 receptor on B cells mediates the maturation of primary follicles into tightly packed secondary follicles with a germinal centre and mantle zone [10]. The germinal centre consists of a dark zone and a light zone. In the dark zone, highly proliferative B cells undergo somatic hypermutation of the immunoglobulin gene, generating high-affinity antibodies. Upon subsequent migration to the light zone, B cells compete for the antigens presented by fDCs. Interactions with T_FH_ cells are critical in determining whether B cells either re-enter the dark zone and undergo further rounds of somatic hypermutation and proliferation, differentiation into a memory B cell or a plasma cell, or apoptosis [11]. If a B cell is positively selected by the T_FH_ cells, signalling through the CD40 receptor, B cell-activating factor (BAFF) and Toll-like receptor (TLR) activates pro-proliferative and pro-survival pathways in the B cell. These pathways converge on the activation of NF-κB [11]. Tight control of transcription factor expression is responsible for exit from the germinal centre and subsequent differentiation of activated B cells into plasma cells or memory cells [11].

Together, these processes lead to the production of high-affinity antibodies that can target pathogens encountered, as well as the generation of long-lived memory B cells and T cells that are able to respond more rapidly to future infection by the same pathogen. In this review, we highlight current knowledge on NK cells within the lymph nodes and discuss potential strategies to improve NK cell function within the lymph nodes of cancer patients.

## 2. NK Cell Distribution and Function in the Lymph Nodes

NK cells are cytotoxic lymphocytes of the innate immune system that occupy peripheral blood, secondary lymphoid organs and other tissues [12]. Peripheral blood NK cells are typically categorised into cytotoxic CD56^dim^ NK cells (~90%) that have higher expression of lytic granule proteins and CD56^bright^ NK cells (~10%) which secrete pro-inflammatory cytokines such as IFNγ and have a more immunomodulatory role [13]. In contrast to the peripheral blood, the majority (75–95%) of NK cells in the secondary lymphoid organs of healthy individuals have a CD56^bright^ phenotype [4,14]. The lymph nodes are also a site of NK cell development as CD34+ haematopoietic stem cells from the lymph nodes can give rise to NK cells in vivo and ex vivo [15,16]. Approximately 70% of lymph node NK cells are negative for CD16, the receptor that mediates antibody-dependent cellular cytotoxicity (ADCC) by binding to the Fc portion of IgG antibodies [14,17]. However, lymph node-derived NK cells can upregulate the expression of CD16 and other activating receptors upon stimulation with IL-2 [4]. NK cells are found in the paracortical T-cell areas and sinuses of normal human lymph nodes [14,18], whereas in mice, they are found in the medulla, paracortex and cortical areas at the B-T cell boundary (Figure 1) [5,19]. The percentages of NK cells reported in the lymph nodes of both humans and mice are summarised in Table 1.

NK cells from the peripheral blood rapidly enter the lymph nodes local to sites of immunisation or infection [5,20,21,22]. Several studies have demonstrated the role of DCs in this recruitment and activation of NK cells. Martin-Fontecha et al. observed a greater than ten-fold increase (~0.3% to ~5%) in the proportion of NK cells in the DC-draining lymph nodes close to the site of immunisation with lipopolysaccharide-matured DCs compared to control lymph nodes without DC infiltrate [20]. In this study, recruitment of NK cells to the lymph node was also found to be important for T_H_1 differentiation, as depletion of NK cells diminished both T-cell proliferation and polarisation to a T_H_1 phenotype. This process was found to be dependent on IFNγ as NK cell-depleted mice reconstituted with NK cells from IFNγ-knock-out (KO) mice were unable to induce T_H_1 differentiation in the DC-draining lymph nodes [20]. However, whether IFNγ is also required for NK cell lymph node recruitment is unclear. In one study, NK cell recruitment to the DC-draining lymph nodes was impaired in IFNγ KO mice [21], whilst another study showed maintenance of NK cell recruitment [20]. Both studies however agreed on the importance of the chemokine receptor CXCR3 expressed on NK cells for lymph node recruitment [20,21], as well as L-selectin and CCR7, which are also important for T-cell lymph node homing [25]. More recently, a DC-based vaccine was shown to induce neutrophil-dependent NK cell recruitment to the DC-draining lymph node, with the depletion of neutrophils in vivo causing impaired NK recruitment and activation [26]. Although no distinct mechanism was elucidated in this study, depletion of neutrophils had little impact on the recruitment and activation of NK cells to the spleens of mice post-DC-vaccination [26], indicating there may be lymph node-specific NK–neutrophil crosstalk driving the recruitment and activation of NK cells following immunisation with a DC-based vaccine.

In addition, priming of peripheral blood and tissue NK cells has been found to be dependent on interactions with DCs within the lymph nodes. Depletion of DCs or inhibition of lymph node recruitment via L-selectin blockade ablated NK cell activation in the blood and tissues [27]. Furthermore, Terme et al. found that NK/DC interactions in the lymph node were impaired by regulatory T cells (Tregs), as Tregs inhibited the expression of CCR5 ligands in the lymph node, which are required for DC recruitment [28]. IL-15Rα is important for DC-dependent NK cell priming and proliferation, and these authors also found that IL-15Rα was upregulated in the absence of Tregs [28]. A two-photon microscopy study found frequent short-lived (<5 min) contacts with DCs were sufficient for NK cell priming in the lymph nodes of mice [29]. NK cells in the lymph nodes had a motile phenotype even at a steady state, and NK cell motility was increased on immunisation with poly I:C, in line with the redistribution of NK cells to modulate immune responses in the lymph nodes.

Reciprocally, NK cells can influence the recruitment and maturation of DCs. NK cells secrete the chemokines XCL1, XCL2 and CCL5, which recruit DCs into the tumour microenvironment [30,31]. Also, NK cell-derived IFNγ can promote DC maturation [32], and NK cells are able to kill immature DCs via engagement of the NK cytotoxicity receptor NKp30 [32,33]. However, these studies were carried out using peripheral blood cells from human donors; therefore, the influence of NK cells on DC maturation in the context of human lymph nodes is yet to be determined. In summary, these data highlight a dynamic flux of NK cells between the lymph nodes and peripheral blood, the role of NK cells in modulating immune response in the lymph nodes, and the potential for their modulation by DCs and Tregs.

NK cells also play a role in regulating B-cell and T-cell immunity in the secondary lymphoid tissue. For example, NK cells can enhance human B-cell antibody production and IgG class switching in vitro via the CD40L:CD40 axis [34] and IFNγ [35]. In contrast, perhaps due to differences between in vitro and in vivo models, NK cells have been implicated in the suppression of germinal centre B cells and T_FH_ cells in murine in vivo models. Rydyznski et al. found that in the absence of NK cells, the numbers of germinal centre B cells and T_FH_ were enhanced in both the spleens and lymph nodes of mice inoculated with lymphocytic choriomeningitis mammarenvirus (LCMV) [36]. This is also evidenced in mice immunised with the non-virus-derived antigen 4-hydroxy-3-nitrophenylacetyl-keyhole limpet hemocyanin [37], which is known to activate both germinal centre B cells and T cells [38]. This effect was dependent on perforin as perforin-KO mice displayed higher numbers of T_FH_ cells and germinal centre B cells regardless of the presence or absence of NK cells [36,37]. NK cell depletion also resulted in reduced viral load and increased expression of antigen-specific IgG1 antibodies [36,37]. An increase in the proportion of BCR sequences that had undergone somatic hypermutation conferring high antigen affinity was also evident [37]. Interestingly, mice depleted of NK cells later during infection failed to show increased germinal centre B-cell numbers compared to mice depleted of NK cells one day before infection [36]. In another study, NK cell depletion 2–3 days before infection with LCMV resulted in lower numbers of splenic T_FH_ cells at day 29 of infection [39], indicating that NK cells may have a suppressive effect on germinal centre immune responses early in infection, but their presence may be required to maintain B-cell and T_FH_ populations long term.

The increase in numbers of germinal centre B cells and humoral immunity with NK cell depletion is dependent on CD4+ T cells. This is most likely due to the role of T-cell-derived signals in the promotion of B-cell survival in the germinal centre, as depletion of CD4+ cells abrogated this effect [36]. However, the NK cell-mediated depletion of T_FH_ is independent of B cells, as the increase in T_FH_ cell number on NK depletion was seen even in B-cell-deficient mice [36]. This indicates that the effect of NK cells on antibody response and germinal centre cellularity is likely predominantly through the regulation of T cells in the secondary lymphoid tissue rather than a direct effect on B cells. Although these studies used a decrease in the number of T_FH_ and B cells as a proxy for NK cell-mediated killing, other studies have demonstrated direct killing of T_FH_ by murine and human NK cells in ex vivo co-culture experiments [40,41].

In addition to affecting germinal centre cellularity, NK cells in secondary lymphoid organs have been studied in the context of clearing viral infections. Single-cell RNA-seq analysis found lymph node NK cells from mice had significantly decreased expression of genes associated with response to viral infection and type I interferons following immunisation with a model of chronic LCMV (Cl13 strain) compared to acute LCMV (Armstrong strain), despite twice the frequency of NK cells in the lymph nodes of Cl13 infected mice [22]. This suggests that NK cells in the lymph nodes may respond to chronic infection but become functionally impaired. Human studies suggest NK cells of the secondary lymphoid tissue are important for the clearance of Epstein–Barr virus (EBV) and human immunodeficiency virus (HIV). Jud et al. demonstrated that IFNγ secreted by human tonsillar NK cells can restrict the growth of EBV-infected autologous B cells [42]. In addition, higher proportions of CXCR5+ NK cells were found in the lymph node B cell follicles of HIV-1 infected patients compared to uninfected controls [43]. These CXCR5+ NK cells were found to have a more active phenotype compared to their CXCR5− counterparts. The presence of CXCR5+ NK cells was also correlated with a lower viral load in the lymph nodes, implicating CXCR5+ NK cells in aiding the clearance of HIV from patient lymph nodes [43]. Another study showed that KLRG1 can act as an immune checkpoint receptor on both peripheral blood and lymph node NK cells in HIV infection, and antibody blockade of KLRG1 was able to enhance the cytotoxicity of peripheral blood NK cells to HIV-infected cells ex vivo [44]. This demonstrates that NK cell function in the lymph node is an important consideration for the development of novel immunotherapies against viral infection.

## 3. NK Cells in the Lymph Nodes of Patients with Haematological Malignancies

Malignant B cells can arise in the germinal centre, and the lymph nodes therefore represent a source of B-cell non-Hodgkin lymphomas (NHLs) [11]. Burkitt lymphoma (BL), diffuse large B-cell lymphoma (DLBCL) and follicular lymphoma (FL) are derived from genomically transformed germinal centre B cells at various stages of germinal centre B-cell differentiation [11]. Mantle cell lymphoma (MCL) originates from mature B cells of the primary lymphoid follicles or mantle zone B cells of secondary lymphoid follicles [45]. Chronic lymphocyte leukaemia (CLL) is thought to originate from pre- or post-germinal centre B cells in the secondary lymphoid tissue [46], and the lymph nodes in CLL patients are characterised by homogenous diffuse sheets of CLL cells. In contrast, acute myeloid leukaemia (AML) and multiple myeloma are typically confined to the bone marrow and spread of these diseases to the lymph nodes is rare [47,48]. Although NK cell-targeting therapies are being exploited for the treatment of B-cell acute lymphoblastic leukaemia (B-ALL) [49,50], we are not aware of any studies to date characterising NK cells in the lymph nodes of B-ALL patients.

The lymph nodes form a critical pro-survival niche in CLL [51], and T_FH_ cells contribute to the survival of NHL and CLL cells through the CD40L:CD40 axis, which activates NF-κB signalling and induces the upregulation of anti-apoptotic proteins [52]. T_FH_ cells also secrete IL-4 [53] which, together with CD40L, can promote the survival and proliferation of lymphoma and CLL cells [54,55,56,57]. These lymph node support signals can also induce resistance to therapies targeting pro-survival pathways [58,59,60,61], and IL-4 may promote an immunosuppressive microenvironment in the lymph nodes by increasing CCL17 expression and thereby recruiting Tregs and more IL-4-secreting T_FH_ cells [62].

NK cells are associated with immune surveillance of B-cell lymphoma, and elevated NK cell counts are associated with improved prognosis of patients [63,64,65]. However, unlike T cells, the role of NK cells in the lymph nodes of B-cell malignancies has been less well studied. The percentage of NK cells reported in the lymph nodes of patients with B-cell malignancies with lymph node involvement is shown in Table 2.

NK cells represent over 10% of the cells in the lymph nodes of DLBCL [66] compared with negligible detection in the lymph nodes of CLL patients [24]. Detailed analysis by spatial transcriptomics has identified NK cells in the lymph node sinuses of NHL patients [18]. Although most NK cells in the lymph nodes of healthy individuals have a CD56^bright^CD16- phenotype [4,14], there is evidence for NK cell cytotoxicity in the lymph nodes of patients with haematological malignancy. For example, in FL lymph node biopsies, Decaup et al. showed that NK cells mediated ADCC against autologous tumour cells ex vivo in response to anti-CD20 rituximab and obinutuzumab [68]. In addition, Enqvist et al. found that over 70% of NK cells in the lymph nodes of FL patients were CD56^dim^CD16+ [67]. A response to anti-CD20 antibodies was also seen in this study in vivo, as granzyme B expression was increased in NK cells from the lymph nodes of FL patients 7 days after treatment with the anti-CD20 antibody rituximab, indicating NK cell activation in lymph nodes [67]. Also, an increase in NK proliferation was observed 7 days after rituximab treatment in both the lymph nodes and peripheral blood as measured by Ki67 expression. NK cell Ki67 expression was higher in FL patient lymph nodes at day 0 compared to healthy tonsil controls, whereas there was no difference in proliferation between patient and healthy control peripheral blood [67]. Overall, this study highlights a phenotypic difference between peripheral blood and lymph node NK cells in patients with FL and indicates that lymph node NK cells in FL patients may be primed for proliferation.

Furthermore, the T_FH_-associated molecules CD40L and IL-4 increase expression of the non-classical MHC-I molecule HLA-E on primary CLL cells ex vivo [70]. In accordance with this, CLL cells that had recently egressed from the lymph nodes were found to have higher HLA-E expression [70]. HLA-E inhibits NK cell function by ligating the inhibitory receptor NKG2A [71], and the increase in HLA-E expression was matched with a decrease in NK cell IFNγ expression and degranulation [70]. This indicates that NK cells may be inhibited in the lymph node microenvironment of CLL via the HLA-E:NKG2A axis. Approximately 50% of NK cells in the lymph nodes of FL patients express NKG2A [67], although this is yet to be characterised in the lymph nodes of CLL patients. In addition, DLBCL cells can evade NK cell lysis by mutations in the gene encoding CD58, the ligand for CD2 on NK cells that can activate NK cell cytotoxicity [72]. However, there is conflicting evidence as to whether NK cells in the DLBCL lymph nodes are beneficial, as high NK cell infiltration has been associated with both better [73,74] and worse [66,75] patient prognosis. Despite this, NK-engaging therapies such as rituximab remain part of the front-line treatment for DLBCL patients [76].

Three-dimensional ex vivo models that attempt to recapitulate the lymph node microenvironment in B-cell malignancies have been developed and these can allow for further elucidation of NK cell activity in haematological malignancies under lymph node-mimicking conditions. These models can be generated from leukaemia or lymphoma cells that are then cultured in ultra-low-attachment plates [77,78,79] or in a collagen matrix [80] to promote 3D spheroid formation under lymph node-mimicking conditions. These conditions include CD40L and BAFF that mimic T-cell-derived ligands, TLR agonists that promote B-cell proliferation, and cytokines such as IL-2, IL-4, IL-15 and IL-21 [77,78,79]. Alternatively, primary DLBCL cells can be co-cultured with fibroblasts and macrophages in 3D to mimic the tumour microenvironment [80]. Three-dimensional FL models derived from patient lymph nodes [68,78] and a patient PBMC-derived model of CLL lymph nodes [79] have been reported to include NK cells; however, studies characterising DLBCL-derived [80] and MCL-derived [77] spheroids did not report NK cell markers in their analyses. In the absence of autologous NK cells, these models would still allow for co-culture studies to model NK cell adoptive transfer strategies for the treatment of B-cell malignancies, as has recently been reported in DLBCL [81].

## 4. NK Cells in the Lymph Nodes of Patients with Solid Tumours

Due to their role in the drainage of lymph from organs, the lymph nodes are often the first site of metastasis of solid tumours [82]. Circulating NK cells have been shown to be important in the control of metastasis in mice [83,84,85], and evasion of NK cytotoxicity is correlated with increased lymph node metastases [86,87]. Beyond this, studies have shown that NK cells within the lymph node niche are important for controlling metastasis of solid tumours. For example, depletion of Tregs with an anti-CD25 antibody was associated with increased activation of NK cells in the lymph node niche of breast cancer-bearing mice and resulted in reduced formation of lymph node metastases [88]. However, the depletion of Tregs had no effect on NK cell activation or metastasis in the lung, suggesting that the effect of Tregs on metastasis may not be systemic [88]. In parallel, lymph nodes in human breast cancer patients had a higher proportion of Tregs and a lower proportion of CD16+CD56^dim^ NK cells compared to lymph nodes from healthy donors [88], indicating that NK cell function in the lymph nodes of cancer patients is likely important in humans.

A study of non-small cell lung cancer patients demonstrated reduced degranulation and cytokine secretion of NK cells in the tumour-draining mediastinal lymph nodes in the thoracic cavity in close proximity to the lungs [89]. This was matched by higher expression of the checkpoint receptor PD-1 and lower expression of NK-activating receptors NKG2D, DNAM and NKp46 on patient lymph node NK cells compared to NK cells from the peripheral blood of patients or healthy controls [89]. Interestingly, removal of the mediastinal lymph nodes in these patients reduced PD-1 expression and enhanced the function of peripheral blood NK cells whilst also being associated with improved survival of patients [89]. The authors concluded that the tumour-draining lymph nodes could be contributing to the exhaustion of peripheral blood NK cells. The lymph nodes may thus represent a source of tumour-stimulated, exhausted NK cells that migrate into the peripheral blood, although further studies are required to confirm this. Another study showed that exhausted NK cells in the mesenteric lymph nodes in a mouse model of colorectal cancer had increased expression of Sirtuin-2, an enzyme involved in negative regulation of metabolism [90]. RNA silencing of Sirtuin-2 expression restored the function of exhausted NK cells in vitro. Interestingly, Sirtuin-2 has also previously been shown to suppress NK cell function in the tumour microenvironment of melanoma [91].

In contrast, the results of other studies suggest that NK cells in tumour-draining lymph nodes are more active than tumour-free lymph nodes. Frazao et al. showed that NK cells in the tumour-draining lymph node of breast cancer patients had higher levels of cytotoxicity against breast cancer cell lines ex vivo as compared to healthy donor lymph node controls [92]. In addition, NK cells from the tumour-draining lymph nodes of metastatic melanoma patients had higher cytotoxicity against autologous melanoma cells and metastatic melanoma cell lines compared to NK cells of tumour-free lymph nodes or healthy donor peripheral blood NK cells [93]. Furthermore, metastatic melanoma patients with a higher ratio of CD57+CD56^dim^ to CD57+CD56^bright^ NK cells in the metastatic lymph nodes showed better survival compared to patients with a lower ratio with the same disease stage [93], suggesting that NK cell cytotoxicity in the metastatic lymph node may be important for disease control. Lymph node tissue-resident NK cells have been described as a distinct CD69+CXCR6+ population [94], and it would be interesting to dissect the phenotype of NK cells in the lymph nodes of cancer patients further by analysing both the lymph node-resident NK cells and NK cells infiltrating from the peripheral blood.

Overall, the expression of NK maturation markers CD16, CD57 and killer-cell immunoglobulin-like receptors on CD56^dim^ NK cells in the tumour-draining lymph nodes [67,92,93] suggests that highly cytotoxic NK cells are present in the lymph nodes of cancer patients and that they may be an attractive target for therapeutic strategies.

## 5. Therapeutic Strategies to Activate NK Cells in the Lymph Nodes of Cancer Patients

**Figure 2:**
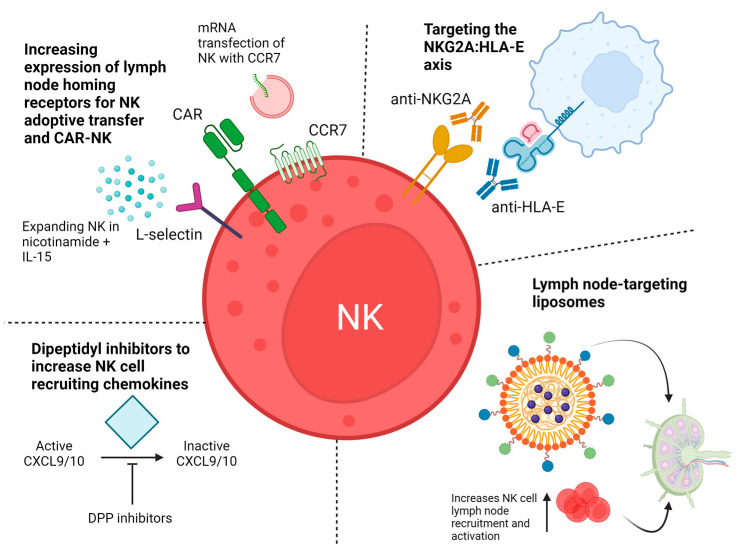
Potential strategies for mobilising and activating NK cells in the lymph nodes of cancer patients. Increasing expression of lymph node-homing receptors via nicotinamide and IL-15 culture or mRNA transfection methods increases lymph node homing of adoptively allogeneic NK cells and could be applied to CAR-NK therapy. Upregulation of HLA-E on malignant B cells by lymph node-associated signals can reduce NK cell function via NKG2A; therefore, blockade of either HLA-E or NKG2A could increase NK cell activation in the lymph nodes. Lymph node-targeting liposomes can increase NK cell recruitment and activation in the lymph nodes. Dipeptidyl peptidase inhibitors prevent cleavage and inactivation of lymph node-recruiting chemokines CXCL9/10, which may be beneficial for increasing lymph node homing. Created with Biorender.com.

NK cell-based therapeutics have an excellent safety record in clinical trials, and various strategies aimed at enhancing their function are currently under pre-clinical and clinical assessment [95]. Due to the critical involvement of the lymph nodes in both haematological malignancies and the metastasis of solid tumours, enhancing NK cell activation in the lymph nodes could represent an effective strategy for enhanced tumour clearance (summarised in Figure 2).

As NK cells represent a relatively small population of lymphocytes in the lymph nodes [4,14,20] (Table 1), one promising strategy is to promote the lymph node homing of allogeneic NK cells in adoptive transfer therapy. NK cell adoptive transfer involves infusion of ex vivo-expanded NK cells derived from healthy human peripheral/cord blood, stem cells or cell lines into a patient and offers the opportunity for genetic modification such as the addition of a chimeric antigen receptor (CAR) construct, or deletion of inhibitory receptors [96,97]. NK cell adoptive transfer strategies are already in clinical trials for both solid and haematological malignancies [95,98]; however, few studies have demonstrated specific trafficking of these cells to tumour-associated lymph nodes. Liu et al. used a K562-based feeder cell to expand NK cells for anti-CD19 CAR-NK therapy, and these showed homing to the lymphoid tissue (spleen, lymph nodes and bone marrow) in NSG mice [99]. Preferential homing of CAR-NK cells to the lymph nodes compared to peripheral blood or bone marrow of patients with B-cell malignancies was also reported for two patients with available lymph node samples [99], although the expression of lymph node-homing molecules such as CCR7 and L-selectin were not characterised in this study.

Interestingly, Cichoki et al. showed that expansion of NK cells with IL-15 and nicotinamide ex vivo induced stable high expression levels of L-selectin, which is important for lymph node trafficking [100]. Adoptive transfer of these cells into NHL patients showed a 74% overall response rate when combined with rituximab in a phase I clinical trial [100]. Alternatively, enhancing CCR7 on the surface of human NK cells by encouraging trogocytosis of the receptor from CCR7+ feeder cells increased lymph node homing in nude mice by 144% [101], and transfection of human NK cells with mRNA encoding CCR7 enhanced migration towards the lymph node-associated cytokine CCL19 in in vitro assays [102]. Moreover, Sanz-Ortega et al. were able to enhance lymph node homing in murine models and ex vivo migration studies by combined transfection of ex vivo-expanded NK cells with L-selectin, CCR7 and CXCR5 [103]. This approach was also demonstrated using ex vivo-expanded NK cells from FL patients, and additional transfection with high-affinity CD16 was able to enhance the cytotoxicity of these NK cells in combination with rituximab [103]. Therefore, expansion methods that retain high expression levels of lymph node-homing molecules or increase surface expression of these molecules via mRNA transfection and other methods may be a promising strategy to enhance lymph node homing of NK cell therapeutics and could synergise with other modalities such as CAR-NK or tumour-targeting antibodies.

CXCR3 is also important for NK cell lymph node homing during an immune reaction and binds the lymph node-associated chemokines CXCL9 and CXCL10 [20,21]. Delivery of dipeptidyl peptidase enzyme inhibitors that block the inactivation of CXCL9 and CXCL10 were found to mobilise CXCR3+ NK cells and T cells to the tumour microenvironment in in vivo models of liver [104] and pancreatic [105] cancer. These inhibitors may also be able to promote the infiltration of NK cells into lymph nodes, although this is yet to be tested.

A recent example targeting autologous NK cell recruitment to the lymph nodes used lymph node-targeting liposomes. Fu et al. designed a liposome-based nano inducer which targets the lymph node and tumour microenvironments via pH-sensitive and matrix-metalloprotease 2 (MMP2)-sensitive ligands [106]. This is relevant because the lymph nodes are known to be acidic [107] and high MMP2 expression has been detected in the tumour-draining lymph nodes of cancer patients [108]. Furthermore, there is greater accumulation of macromolecules of 10–100 nm in the lymph nodes as lymphatic capillaries are more permeable than peripheral blood capillaries [109,110]. Temperature-sensitive liposomes were loaded with the photothermal agent IR780 that converts light to heat energy upon light irradiation. This had a dual function: to kill cancer cells directly and break down the liposome to release IDO1 inhibitor 1-MT and inhibit Treg recruitment. Activation of cytotoxic T cells and NK cells was also made possible by expressing IL-15 on the surface of the liposome. The authors showed that delivery of this construct increased the abundance of NK cells in the lymph nodes from ~1.6% to ~16% in a mouse model of melanoma, whilst the abundance of Tregs decreased. This correlated with improved survival and a reduction in tumour size. Maulhardt et al. also showed NK cell accumulation in the lymph nodes in response to combined anti-PD-1- and paclitaxel-loaded microparticles relative to vehicle and isotype controls [111], although the proportion of NK cells in the lymph nodes more modestly increased from ~0.75 to ~1.2%.

Liposomes coated with both the pro-apoptotic protein TRAIL and antibodies targeting the mouse NK cell surface molecule CD57 combined with NK cells induced greater apoptosis of cancer cell lines compared to NK cells or liposomes alone in an in vitro 3D model mimicking the lymph node architecture [112]. The authors went on to develop a similar TRAIL-coated liposome targeting NK1.1 [113] and CD335 (NKp46) [114]. This strategy reduced tumour burden in the lymph nodes in a mouse model of metastatic colorectal cancer. Anti-CD335 liposomes were retained in the inguinal lymph nodes of the mice for at least 4 days when injected intravenously, whereas the majority of these liposomes injected intraperitoneally were lost by day 4, indicating that the delivery route of liposomes is important for lymph node retention [114]. Although this strategy has only been tested in the context of solid tumour metastasis to the lymph nodes, malignant B cells are also susceptible to TRAIL-induced apoptosis [115], and this could represent an attractive strategy for targeting B-cell lymphoma as well.

Lymph node-associated signals CD40L and IL-4 increase HLA-E expression on the surface of primary CLL cells [70]. The upregulation of NKG2A on NK cells expanded ex vivo [71] and on NK cells in the tumour-positive lymph nodes [67,92] implies that the NKG2A:HLA-E axis could be targeted to improve NK cell function in the lymph nodes of patients with B-cell malignancies. For example, the first-in-class exportin-1 inhibitor selinexor, which is FDA-approved for the treatment of multiple myeloma and DLBCL, downregulates the surface expression of HLA-E on both lymphoma cell lines [116] and primary CLL cells [70], resulting in improved NK cell function against these targets. Additionally, CRISPR-KO of NKG2A can improve tumour clearance by anti-CD133 CAR-NK cells relative to NKG2A-WT CAR-NK cells in a mouse model of acute myeloid leukaemia [117], and this strategy may be effective in potentiating the function of adoptively transferred NK cells in the lymph nodes of patients with B-cell malignancies. Direct disruption of the NKG2A:HLA-E axis is also achievable using antibodies. For example, anti-HLA-E antibodies have been shown to enhance the function of NK cells in vitro but are still in pre-clinical studies [118,119]. In contrast, the anti-NKG2A antibody monalizumab is currently in multiple clinical trials for the treatment of solid tumours (NCT04307329, NCT02671435, NCT05221840, NCT05903092, NCT03801902) and may also have utility in boosting NK function against malignant B cells within the lymph nodes.

Interestingly, NKG2A blockade may also be effective in preventing metastasis of solid tumours. NK cells were the predominant immune cell interacting with circulating tumour cells in blood biopsies of metastatic pancreatic ductal adenocarcinoma patients, and in a transcriptomic analysis, the HLA-E:NKG2A axis was the most enriched interaction between these cells [87]. Indeed, early treatment with an NKG2A-blocking antibody was able to reduce lung metastasis in a mouse model of metastatic pancreatic cancer; however, delivery of NKG2A blockade 3 or 5 days after tumour inoculation still resulted in significant metastasis by day 15 [87]. However, whether NKG2A blockade would be effective in preventing lymph node metastases is yet to be determined.

## 6. Conclusions

NK cells in the lymph nodes have an important role in regulating germinal centre cellularity and lymph node homeostasis during infection. Evasion of NK cell cytotoxicity in the lymph nodes has also been shown to be associated with increased metastasis of certain solid tumours and is correlated with worse patient outcomes. In haematological malignancies, NK cells are present in the lymph nodes and have been shown to respond to tumour-targeting antibodies. Potential therapeutic strategies to mobilise NK cells to the lymph nodes include the adoptive transfer of NK cells expressing high levels of lymph node-homing receptors, increasing NK cell-recruiting chemokines and use of lymph node-targeting liposomes. Blockade of immune checkpoints such as NKG2A may also be effective in enhancing NK cell activation against tumour cells within the lymph nodes. Ultimately, the development of strategies that can overcome the immunosuppressive microenvironment and improve tumour clearance from the lymph nodes holds great potential to improve the survival of patients with cancer.

## Figures and Tables

**Table 1:** NK cell percentages (%) reported in the lymph nodes.

	Lymph Node State	% NK Cells	Reference(s)
Mouse	Resting	~0.2–0.5	[5,20,21]
	DC-draining	~5–12	[20,21]
	Viral infection	1.5, 4–6	[5,22]
Rat	Resting	~2	[23]
Human	Resting	~1–7	[4,14]
	Normal/non-cancer	~1	[24]

**Table 2:** NK cell percentage (%) in the lymph node microenvironment of B-cell malignancies.

	% NK Cells	Reference(s)
Diffuse large B-cell lymphoma	11–12	[66]
Follicular lymphoma	0.5–1	[67,68]
Burkitt lymphoma	<5	[69]
Chronic lymphocytic leukaemia	Negligible	[24]
